# The Effects of Watermelon Juice on Muscle Hypertrophy, Exercise Performance, and Muscle Soreness in Non‐Athlete Men Undergoing Endurance Training: A Randomized Controlled Trial

**DOI:** 10.1002/fsn3.71070

**Published:** 2025-10-12

**Authors:** Mostafa Norouzzadeh, Behrooz Gholami, Melika Samari, Hadi Atarod, Fatemeh Sadat Hosseini‐Baharanchi, Seyedeh Tayebeh Rahideh

**Affiliations:** ^1^ Department of Nutrition, School of Public Health Iran University of Medical Sciences Tehran Iran; ^2^ The Persian Gulf Tropical Medicine Research Center, The Persian Gulf Biomedical Sciences Research Institute Bushehr University of Medical Sciences Bushehr Iran; ^3^ Medical Physics Department Iran University of Medical Sciences Tehran Iran; ^4^ Department of Sport Sciences, Science and Research Branch Islamic Azad University Tehran Iran; ^5^ Minimally Invasive Surgery Research Center & Department of Biostatistics, School of Public Health Iran University of Medical Sciences Tehran Iran

**Keywords:** citrullus, dietary supplements, endurance training, exercise performance, muscle hypertrophy

## Abstract

In the current study, we aimed to assess the impact of watermelon juice as a functional drink on muscle soreness, exercise performance, and muscle hypertrophy. This open‐labeled randomized controlled trial was conducted on 42 non‐athlete men for 8 weeks. The participants were randomized into two equal groups: the intervention group received 710 mL of watermelon juice, and the control group received a calorie‐matched placebo 1 h before exercise. Also, both groups received an endurance exercise plan 3 days a week and were recommended to maintain their dietary routines throughout the study period. At the end of the first week, exercise performance was evaluated, and participants were asked to complete the muscle soreness chart immediately and 24 h after the exercise. Finally, muscle hypertrophy, including the thickness of the pectoralis major and rectus femoris muscles, was measured by ultrasound at the end of the study. No significant differences were observed in exercise performance between groups. However, the intervention group showed a significant increase in the thickness of the pectoralis major (MD: 2.00, 95% confidence interval: 0.87, 3.13) and rectus femoris (MD: 1.70, 95% confidence interval: 0.72, 1.68) muscles and reported lower muscle soreness 24 h post‐exercise compared to the control group (*p* = 0.045). In conclusion, watermelon juice may benefit individuals engaged in regular exercise by optimizing muscle repair and hypertrophic responses. Further research is warranted to confirm its effects on exercise performance across diverse conditions.

**Trial Registration:** This trial was registered on the Iranian Registry of Clinical Trials (IRCT) under registration number IRCT20191105045340N1 on 15/12/2019 (https://irct.behdasht.gov.ir/).

## Graphical abstract

This open‐labelled randomized controlled trial investigates how incorporating watermelon juice into an endurance exercise regimen affects muscle hypertrophy, exercise performance, and muscle soreness. Short supplementation durations and limited dosage exploration have often constrained previous studies. In this study, we administered 710 ml of watermelon juice (approximately 1.65 g L‐citrulline) over a longer period (8 weeks) compared to previous studies. The findings revealed reduced muscle soreness and increased thickness in muscles engaged in the exercise plan. Overall, our results supported watermelon juice as an ergogenic aid.

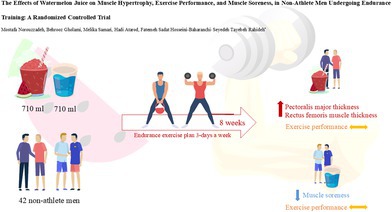

## 1 Background

Scientists are working towards developing a highly effective method to optimize exercise performance (Yan et al. 2016). This involves addressing the effects of high‐intensity exercise, including increased glycolysis, blood lactate accumulation, fatigue, muscle soreness, local inflammation, and oxidative stress (MacIntyre et al. 1996; Mutch and Banister 1983; Zerba et al. 1990). Endurance activities cause muscle damage, which leads to decreased performance (Callis et al. 1991). Consequently, ergogenic aids improve exercise performance by promoting blood flow and facilitating nutrient delivery to active muscles (Cribb and Hayes 2006; McMahon and Jenkins 2002).

There is an interest in utilizing fruits and fruit juices to improve both exercise performance and overall health (Shanely et al. 2016). Fruits, a rich source of carbohydrates and antioxidants, play a crucial role in sustaining blood glucose levels during endurance activities, reducing post‐exercise inflammation, enhancing antioxidant capability, and notably prolonging time to exhaustion (Murdoch et al. 1993; Nieman et al. 2015).

Watermelon ( *Citrullus lanatus* ) belongs to the Cucurbitaceae family and contains approximately 91% water and about 6% sugar by weight (USDA‐NRCS 2014). Watermelon is a substantial source of bioavailable compounds such as lycopene, carotenoids, glutathione, and L‐citrulline (Rimando and Perkins‐Veazie 2005). Lycopene contributes to watermelon's red color and is a potent antioxidant (Stahl and Sies 2003). Additionally, L‐citrulline, can convert into L‐arginine, augment nitric oxide (NO) synthesis, and thereby promote vasodilation (Wu and Morris Jr. 1998). Studies have shown that L‐Citrulline supplementation is more effective than L‐arginine supplementation in boosting plasma L‐arginine concentrations and enhancing NO bioactivity (Schwedhelm et al. 2008). Moreover, combining L‐citrulline with glutathione may further amplify NO production, suggesting that watermelon juice could be a more effective dietary intervention (Davis et al. 2013; McKinley‐Barnard et al. 2015).

Considering limited available data, previous studies have generally indicated the effectiveness of watermelon supplementation, although there is a controversy among their findings (Bailey et al. 2016; Cutrufello et al. 2015; Gonzalez et al. 2022; Harnden et al. 2023; Martínez‐Sánchez, Alacid, et al. 2017; Martínez‐Sánchez, Ramos‐Campo, et al. 2017; Tarazona‐Díaz et al. 2013). Studies have indicated that supplementation with watermelon juice before exercise in male runners resulted in improved exercise strength, reduced perceived exertion and muscle soreness after exercise, and decreased plasma lactate concentrations in resistance‐trained males (Martínez‐Sánchez, Alacid, et al. 2017; Martínez‐Sánchez, Ramos‐Campo, et al. 2017). Tarazona‐Díaz et al. showed that acute watermelon juice (1.2 g of L‐citrulline) or enriched watermelon juice (6 g L‐citrulline) supplementation reduced muscle soreness but did not improve performance in untrained individuals (Tarazona‐Díaz et al. 2013). Gonzalez et al., indicated that short‐term watermelon supplementation did not enhance exercise performance or muscle oxygenation parameters in resistance‐trained men (Gonzalez et al. 2022). Similarly, acute consumption of watermelon juice did not improve aerobic performance or time to exhaustion in healthy male individuals (Cutrufello et al. 2015). Although watermelon juice did not improve endurance performance in severe‐intensity exercise, a study by Bailey et al. (Bailey et al. 2016), recommends to further research with higher doses and longer follow‐up (Harnden et al. 2023).

Therefore, this randomized placebo‐controlled trial aimed to evaluate the effect of watermelon juice in combination with endurance training on post‐exercise muscle soreness, exercise performance, and muscle hypertrophy in non‐athletic men over 8 weeks.

## 2 Materials and Methods

### 2.1 Design

This study was an 8‐week parallel‐group, open‐label, randomized controlled trial (RCT) designed to evaluate the effects of watermelon juice supplementation combined with an endurance exercise plan on muscle soreness, exercise performance, and muscle hypertrophy. The study was reported following the CONSORT guidelines for reporting parallel‐group randomized trials (Schulz et al. 2010). The complete CONSORT checklist is provided as a Data S1.

### 2.2 Participants

Participants were student residents of the dormitories at Iran University of Medical Sciences (IUMS), Tehran, Iran. They were healthy males aged 18–35 years with a body mass index (BMI) of 20–25 kg/m^2^ and a daily caffeine intake of less than 250 mg. Exclusion criteria included using sports or dietary supplements, metabolism‐stimulating drugs, or a history of physical fitness‐affecting diseases. Additional exclusion criteria were smoking, engaging in bodybuilding activities within the past 6 months, noncompliance with the study protocol, withdrawal of consent, or development of a medical condition affecting exercise ability. Any side effects from the supplementation were documented, and affected participants were withdrawn from the study. Finally, eligible individuals were provided detailed information about the study and gave written informed consent before participation.

### 2.3 Randomization and Blinding

Eligible participants were randomly assigned to either the intervention or control group using a randomization list generated by a biostatistician with the blockrand package in R (version 3.3.4), employing a four‐block size. To reduce the risk of outcome reporting bias, participants in the intervention group were housed in one randomly selected dormitory, while participants in the control group were housed in another. Both dormitories were matched to ensure comparable gym facilities, environmental conditions, and other potential confounding factors.

All participants were instructed not to engage in strenuous physical activity or consume caffeine 24 h before the measurements. Moreover, they were advised to maintain their usual physical and dietary routines throughout the study period. The height and weight of each participant were measured at the beginning of the study using standard procedures (Heiat et al. 2001). In addition, the 24‐h dietary recall for 3 days (two weekdays and one weekend) and validated International Physical Activity Questionnaires (IPAQ) for the Iranian population were completed at the beginning and the end of the study (Lee et al. 2011; Moghaddam et al. 2012). All enrolled participants demonstrated full adherence to the study protocol (100%). This high level of compliance was achieved by having a study colleague (BGH and MN) present at the gym during each session to supervise and ensure consumption of the assigned beverage directly.

### 2.4 Interventions

Participants were randomly assigned to two groups: the watermelon juice group and the isocaloric drink group. Both groups followed an 8‐week endurance training program designed to promote muscle hypertrophy, with sessions conducted 3 days/week under the guidance of a coach. The intervention group received fresh watermelon juice (approximately 1.65 g of L‐citrulline per serving), while the control group received an isocaloric drink (Table 1). Both drinks were served in 710 mL shakers and consumed within 30 min, starting 1 h before exercise (Cutrufello et al. 2015; Tarazona‐Díaz et al. 2013). The isocaloric drink matched the watermelon juice in volume, caloric content, and carbohydrate composition and was flavored with lemon juice and sugar to ensure palatability (Cutrufello et al. 2015). Exercise performance and muscle soreness were evaluated after the first week of intervention. After 8 weeks, muscle hypertrophy was assessed using validated imaging techniques.

**TABLE 1:** The composition of selected nutrients in 100 g of watermelon (*Crimson sweet*).

Nutrients	Content	Nutrients	Content
Energy	30 Kcal	Vitamin B6	0.045 mg
Carbohydrate	7.55 g	Choline	4.1 mg
Fiber	0.4 g	Vitamin C	8.1 mg
Fat	0.15 g	Beta‐caroten	0.303 mg
Protein	0.61 g	Lycopen	4.53 mg
Water	91.4 g	Arginine	59 mg
Thiamin (B1)	0.033 mg	Pottasium	112 mg
Riboflavin (B2)	0.021 mg	Sodium	1 mg
Niacin (B3)	0.178 mg	Calcium	7 mg
Pantothenic acid (B5)	0.221 mg	Magnesium	10 mg

### 2.5 Outcomes

#### 2.5.1 Primary Outcomes

##### 2.5.1.1 Muscle Hypertrophy Measurements

Participants were introduced to the radiology clinic to measure their muscle thickness. The thickness of the pectoralis major and rectus femoris muscles (both dominant sides of the body) was measured by ultrasound. To measure the thickness of the rectus femoris, a hypothetical line was first drawn from the upper tip of the patella to the upper anterior iliac spine. Then, in the middle of this line, a radiologist measured and reported the muscle thickness in millimeters (mm) using a Samsung ws80 ultrasound device and a 38 mm probe. Also, the thickness of the pectoralis major in the midclavicular line in the third intercostal space was measured using the same protocol.

##### 2.5.1.2 Exercise Performance Test

To evaluate exercise performance, we assessed exercise strength and endurance. Exercise strength was measured at 100% repetition maximum (RM). The process involved three steps. Firstly, individuals warmed up by completing 5–10 repetitions of the exercise at 40%–60% of their estimated 100% RM. Then, after a minute of rest and stretching, they performed 3–5 repetitions of the exercise at 60%–80% of the estimated 100% RM. Finally, the weights were increased conservatively after 3–5 min of rest if they could successfully lift weights. This process was repeated until the person was unable to lift weights (Gibson et al. 2024). After calculating the 1RM, exercise endurance was measured by counting the repetitions at 70% RM (Gibson et al. 2024).

##### 2.5.1.3 Muscle Soreness Assessment

Participants were asked to assess the muscle soreness they experienced using a visual analog chart (VAS) immediately after training and 24 h after training during the first week after each training session (Aaron et al. 2017).

### 2.6 Sample Size Calculation

The sample size was calculated based on the power of 80% and the probability of type I error of 5%. For exercise performance, a minimum of 19 participants was required based on a mean difference of 1.1 repetitions (Gonzalez et al. 2018) and muscle hypertrophy (*n* = 12) by a mean difference of 4% (Lowery et al. 2013), respectively. The final sample size was considered to be 10% attrition, with 21 individuals for each treatment group.

### 2.7 Statistical Analysis

The data were analyzed utilizing the IBM SPSS Statistics (Version 26.0, IBM Corp., Armonk, NY, USA). The data are presented as mean ± standard deviation (SD). The normal distribution of the data was ascertained through a graphical approach and the Kolmogorov–Smirnov test. An independent *t*‐test and chi‐square test were used to compare baseline characteristics. All final analyses were conducted according to the intention‐to‐treat (ITT) principle, including all enrolled and randomized participants. Missing values for biochemical variables were addressed using multiple imputation. Specifically, observed baseline variables were used to impute missing outcome values via a linear regression model. The significance level was set at a *p*‐value < 0.05.

## 3 Results

**FIGURE 1:**
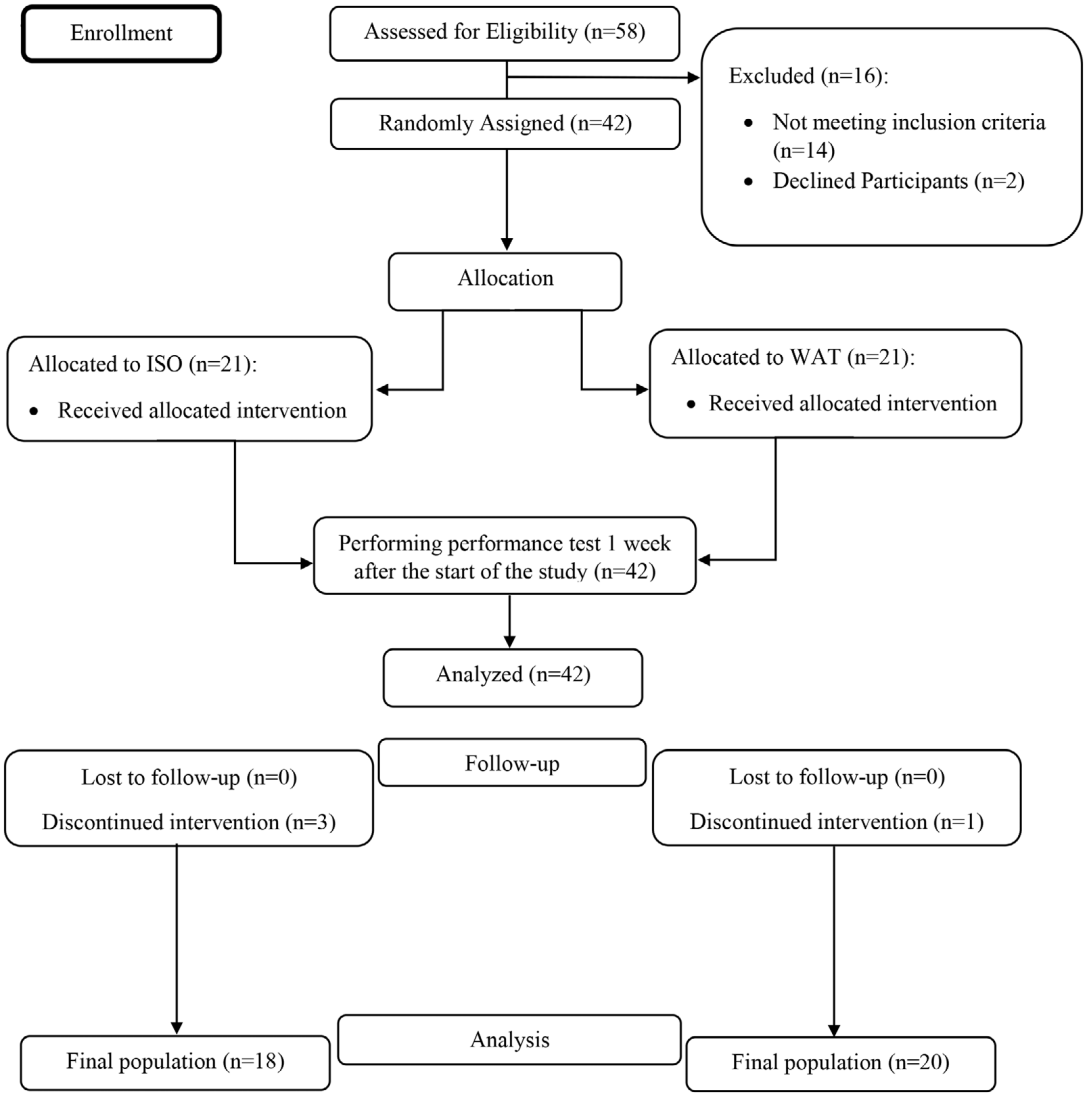
Follow‐up diagram for study participants in watermelon juice (WAT) and control group (ISO) groups.

Participant recruitment was conducted between December 2019 and May 2020 (COVID‐19 period). As shown in Figure 1, out of the initial 58 eligible individuals, 42 were ultimately enrolled in the study. Assessment of muscle soreness and exercise performance was conducted during the first week of the study, involving 42 participants. However, four participants were subsequently excluded from the study due to relocation (*n* = 1), not adhering to the study protocol (*n* = 2), and owing to illness during the study period (*n* = 1). Consequently, muscle hypertrophy measurements were performed on the remaining 38 participants, comprising 20 participants in the intervention and 18 participants in the control group.

No significant difference was observed in the baseline demographic and anthropometric characteristics of the participants, including age, height, weight, body mass index, and physical activity levels, across both the intervention and control groups. Additionally, the two study groups had no significant difference in energy, carbohydrate, protein, and fat intake (Table 2).

**TABLE 2:** Baseline characteristics of the study participants*.

Characteristics	Treatment group	Control group	*p*
	Watermelon juice + endurance exercise plan (*n* = 20)	Placebo + endurance exercise plan (*n* = 18)	
Age (year)	26.62 ± 5.14	26.19 ± 5.63	0.798
Height (cm)	175.67 ± 5.19	176.62 ± 5.03	0.550
Weight (kg)	74.93 ± 8.01	76.24 ± 9.12	0.624
Body mass index (kg/m^2^)	24.22 ± 0.19	24.50 ± 0.27	0.580
Physical Activity (MET − minutes/week)	2497.67 ± 327.98	2691.59 ± 326.96	0.597
Energy (kcal/d)	2655.24 ± 831.87	2926.90 ± 963.36	0.334
Protein intake (g/d)	105.990 ± 30.34	108.76 ± 26.68	0.748
Carbohydrate intake (g/d)	382.06 ± 122.59	460.76 ± 207.52	0.142
Fat intake (g/d)	80.25 ± 46.42	73.92 ± 24.44	0.584

* Data is presented as mean ± standard deviation. The *p*‐values for age, height, weight, energy, protein, carbohydrate, and fat were calculated using the Independent *t*‐test, and the Mann–Whitney *U*‐test was used for body mass index and physical activity.

Table 3 outlines the impact of watermelon juice on the control group's exercise performance. Notably, exercise strength significantly increased in both the intervention and control groups following 1 week of supplementation (*p* = 0.001). However, the intervention and control groups observed no significant difference in exercise strength. The intervention group showed a slightly higher exercise endurance improvement than the control group; however, this discrepancy was inconclusive and lacked statistical significance.

**TABLE 3:** Effect of watermelon juice versus control group on exercise performance in the first week of the study*.

Exercise strength (100% RM)				Exercise endurance (70% RM)			
	Intervention (*n* = 21)	Control (*n* = 21)			Intervention (*n* = 21)	Control (*n* = 21)	
Bench press (kg)			MD (95% CI)	Bench press (repetitions)			MD (95% CI)
Before	42.0 (4.98)	41.9 (4.93)	0.10 (−1.86, 2.06)	Before	5.52 (0.98)	6.71 (1.64)	0.52 (−0.03, 1.07)
After	44.4 (4.93)	44.2 (5.48)		After	5.71 (1.00)	6.38 (1.28)	
Change	2.40 (3.13)	2.30 (3.33)		Change	1.19 (1.03)	0.67 (0.76)	
Leg press (kg)				Leg press (repetitions)			
Before	58.3 (8.19)	59.2 (7.71)	1.00 (−2.16, 4.16)	Before	6.10 (1.26)	6.05 (1.39)	0.28 (−0.41, 0.97)
After	61.6 (9.03)	61.5 (7.92)		After	7.19 (2.01)	6.86 (1.68)	
Change	3.30 (5.50)	2.30 (4.94)		Change	1.09 (1.25)	0.81 (1.00)	

Abbreviations: CI, confidence interval; MD, mean difference; RM, repetition maximum.

* Data are presented as mean (standard deviation) and mean difference (95% CI).

Table 4 displays the thickness of the pectoralis major and rectus femoris muscles before and after the intervention. Following 8 weeks of supplementation, the thickness of the pectoralis major muscle significantly increased in both the intervention and control groups. Additionally, the thickness of the rectus femoris muscle increased in both study groups, with a notably greater increase observed in the intervention group compared to the control group.

**TABLE 4:** Effect of watermelon juice versus control group on muscle hypertrophy after the eighth week of the study*.

Muscle hypertrophy			
	Intervention (*n* = 21)	Control (*n* = 21)	MD (95% CI)
Pectoralis major thickness (mm)			
Before	21.3 (2.78)	21.3 (2.67)	2.00 (0.87, 3.13)
After	24.9 (3.19)	22.9 (2.35)	
Change	3.60 (1.92)	1.60 (1.61)	
Rectus femoris thickness (mm)			
Before	23.0 (2.11)	22.6 (1.90)	1.70 (0.72, 2.68)
After	26.6 (2.94)	24.5 (2.11)	
Change	3.60 (1.78)	1.90 (1.28)	

Abbreviations: CI, confidence interval; MD, mean difference.

* Data are presented as means (standard deviations) and mean differences (95% CI).

**FIGURE 2:**
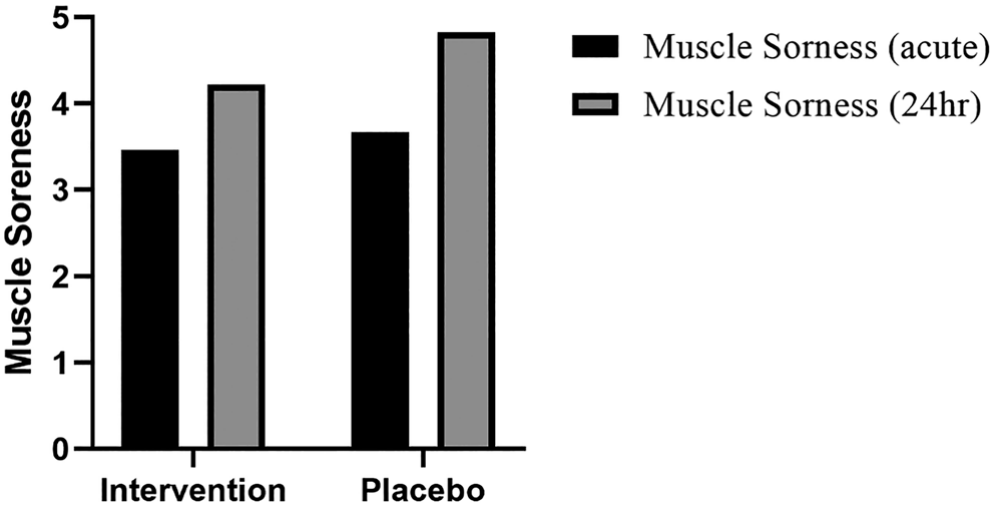
Comparison between muscle soreness score, in acute (*p* = 0.581) and 24‐h (*p* = 0.045) post‐exercise periods in intervention and control groups.

Figure 2 represents the differences between muscle soreness scores between the intervention and control groups at acute and 24‐h post‐exercise time points. There was no significant difference in acute muscle soreness following exercise between the intervention (3.46 ± 0.98) and control (3.67 ± 1.39) groups. However, the muscle soreness 24 h after exercise was significantly (*p* = 0.045) lower in the intervention group (4.22 ± 0.78) than in the control group (4.83 ± 0.82). Notably, we did not observe any side effects that led to study discontinuation in both intervention and control groups.

## 4 Discussion

Overall, while no notable variance in exercise performance was observed between the intervention and control groups following 1 week of supplementation, outcomes in the eighth week revealed a greater thickness in the pectoralis major and rectus femoris muscles among participants in the intervention group compared to the control group. Our study findings suggest that watermelon juice supplementation could effectively alleviate post‐endurance exercise muscle soreness.

Previous studies attribute the favorable effects of watermelon on exercise performance to its food matrix components (Gonzalez and Trexler 2020). Watermelon is rich in antioxidants, potassium, and citrulline (Edwards et al. 2003). Specifically, a single 286‐g serving of watermelon contains 0.429 g of L‐citrulline and 0.135 g of L‐arginine (Shanely et al. 2016). Watermelon contains a notable amount of antioxidants like glutathione (Edwards et al. 2003). When combined with citrulline, glutathione improves NO synthesis more effectively than citrulline alone (McKinley‐Barnard et al. 2015). Several studies have demonstrated that watermelon juice can increase the circulating arginine pool and activate NO synthase more than arginine (Bailey et al. 2016; Collins et al. 2007). The ratio of fructose to glucose in watermelon is 0.55, as per the study conducted by Shanely et al. (2016). Although the gut absorption rate of exogenous fructose is relatively low compared to glucose, watermelon supplementation has been demonstrated to be an effective means of meeting the energy requirements of vigorous endurance cycling (Shanely et al. 2016). Additionally, watermelon puree is comparable to a 6% carbohydrate beverage in enhancing endurance exercise performance. Furthermore, it offers the added benefit of increased satiety and enhanced antioxidant capacity due to its lycopene, vitamin A, and vitamin C content (Shanely et al. 2016).

According to our knowledge, this study represents the first inquiry into the effects of watermelon juice supplementation on muscle hypertrophy. Our results indicate a significant increase in muscle thickness, particularly in the pectoralis major and rectus femoris muscles. While we observed a substantial impact of watermelon on muscle hypertrophy, the certainty of this impact remains unclear due to the extensive range of confidence intervals. The precise physiological mechanisms that underpin this effect remain unclear, but previous studies have shown that a one‐week refeeding period with a diet enriched in L‐citrulline (at a dose of 5 g/kg/day) led to a marked increase in the absolute rate of muscle protein synthesis (Osowska et al. 2006).

Furthermore, our findings indicated a significant reduction in muscle soreness 24 h post‐exercise. Consistent with our results, Martínez‐Sánchez et al. found that pre‐exercise supplementation with watermelon juice in healthy male runners resulted in a notable decrease in post‐exercise rating of perceived exertion and muscle soreness (Martínez‐Sánchez, Alacid, et al. 2017). A recent study highlighted that supplementation with watermelon juice, given 1 h before exercise in resistance‐trained males, decreased the perception of muscle soreness during the 24–72 h post‐exercise period (Martínez‐Sánchez, Ramos‐Campo, et al. 2017). Moreover, it sustained lower plasma lactate concentrations following exhaustive exercise (Martínez‐Sánchez, Ramos‐Campo, et al. 2017). Another study showed that acute supplementation with watermelon juice, containing either 1.2 g or 6 g of L‐citrulline, reduced moderate muscle soreness in healthy individuals who participated in high‐intensity exercise intervals (Tarazona‐Díaz et al. 2013). Conversely, the intake of watermelon juice containing roughly 1 g of L‐citrulline did not yield any improvements in the duration of exhaustion during a graded exercise test (Cutrufello et al. 2015). Furthermore, Bailey et al. conducted a study where subjects were supplemented with watermelon juice for 16 days, providing 3.4 g of L‐citrulline per day. They reported no increase in exhaustion duration during high‐intensity exercise (Bailey et al. 2016). Nonetheless, these studies were of relatively short duration. A recent systematic review recommends further research with higher continuous doses over 7 days to potentially achieve favorable outcomes (Harnden et al. 2023).

The findings of our study revealed that the intervention group exhibited potential benefits in exercise performance. However, no significant difference was observed in exercise performance between the intervention and control groups. Our objective was to evaluate exercise performance independently of muscle hypertrophy, and thus, we measured exercise performance after 1 week of supplementation. Nevertheless, the administration of watermelon juice may require a more extended duration to yield a significant effect on exercise performance. Cutrufello et al. (2015) conducted a study to evaluate the effects of L‐citrulline on the aerobic performance of healthy male individuals. The study involved the administration of a single dose of L‐citrulline (6 g) and watermelon juice (containing approximately 1.0 g L‐citrulline) as a pre‐exercise supplement. The results showed no significant changes in the aerobic performance of the participants (Cutrufello et al. 2015). Similarly, Gonzalez et al. conducted a cross‐over trial involving 15 resistance‐trained men. The trial aimed to examine the effects of watermelon juice concentrate, containing 2.2 g of L‐citrulline per day, on the exercise performance of the participants. The participants were given watermelon juice concentrate for 7 days before performing an exercise protocol. The study revealed no significant changes in the isometric force production, bench press performance, blood vessel diameter, or muscle oxygenation parameters compared to a control (Gonzalez et al. 2022). Studies have attributed the lack of effect to two factors—the short duration of supplementation and the slightly lower daily dose of L‐citrulline administered. The daily dose of L‐citrulline administered fell below the proposed minimum effective dose of 3 g. This minimum effective dose has been reported in existing literature for improving resistance exercise performance with isolated L‐citrulline (Gonzalez et al. 2022).

Watermelon, like other functional foods, exhibits its effects through the unique combination of its nutrients and their interactions. Key components such as citrulline, glutathione, and arginine reduce muscle soreness and enhance muscle hypertrophy. In a randomized controlled trial, daily watermelon supplementation over 2 weeks led to elevated post‐exercise blood levels of essential watermelon nutrients, including L‐citrulline and L‐arginine, increased antioxidant capacity, and higher total nitrate levels (Shanely et al. 2016). Watermelon juice is a rich source of L‐citrulline, a non‐essential amino acid that has been found to contain approximately 2.33 g per liter of unpasteurized juice (Tarazona‐Díaz et al. 2013). L‐citrulline plays a vital role in reducing lactic acid accumulation, leading to improved resistance exercise performance until exhaustion (Callis et al. 1991). L‐citrulline is synthesized indirectly from arginine via ornithine and is involved in the sequestration of ammonia within the urea cycle. Notably, citrulline converts back to arginine via arginosuccinate in a dual enzymatic process (Harnden et al. 2023). Furthermore, NO is synthesized from L‐arginine by tetrahydrobiopterin (BH4)‐dependent NO synthase (Wu and Morris Jr. 1998). This potent vasodilator facilitates increased blood flow and mitochondrial respiration, especially during exercise (Bescós et al. 2012; Suzuki et al. 2016). Vincellette et al. demonstrated that daily watermelon juice supplementation for 2 weeks augmented flow‐mediated dilation and microvascular function (Vincellette et al. 2021). Additionally, NO enhances muscle contractility, repair, blood flow, glucose uptake, and resistance exercise performance (Glenn et al. 2017; Petrovic et al. 2008). NO may also optimize the coupling between ATP hydrolysis and exercise efficiency, hence reducing the ATP cost of force generation and leading to enhanced muscle force generation (Jones et al. 2018). Besides, NO promotes the translocation of glucose transporter type 4 (GLUT4), augmenting glucose flux, which could potentially enhance exercise performance and muscle hypertrophy (Bradley et al. 1999). However, reactive oxygen species (ROS) can deactivate NO within tissues. Antioxidants present in watermelon juice may maintain NO biological functions by protecting it from oxidative damage (Ignarro 2000).

Several alternative mechanisms have been proposed to mitigate muscle soreness and improve muscle hypertrophy. One of these mechanisms is the usage of citrulline, which has been shown to buffer ammonia levels through the urea cycle (Bendahan et al. 2002; Martínez‐Sánchez, Ramos‐Campo, et al. 2017). Accumulation of ammonia is a known contributor to muscular fatigue, which can have a significant impact on endurance performance (Chen et al. 2020; Hellsten et al. 1999). In addition, elevated ammonia levels promote anaerobic glycolysis, leading to increased blood lactate levels (Mutch and Banister 1983). Thus, supplementation with watermelon, which is rich in citrulline, is hypothesized to reduce ammonia levels and enhance aerobic respiration (Bendahan et al. 2002; Martínez‐Sánchez, Ramos‐Campo, et al. 2017). Additionally, studies proposed that augmenting NO availability, combined with the potential antioxidant properties of other bioactive compounds present in watermelon juice, may lead to improved muscle oxygenation and accelerated VO_2max_ kinetics (Bailey et al. 2009; Vincellette et al. 2021). The rate of increase in VO_2max_ is restrained by the capacity to supply oxygen to active muscles at the onset of exercise (Xu and Rhodes 1999). A study conducted by Ashley et al. (2018) found that a seven‐day supplementation of L‐citrulline enhanced VO_2max_ kinetics during walking in males. Similarly, a seven‐day supplementation of 6 g of citrulline was shown by Bailey et al. to lead to overall faster VO_2max_ kinetics during severe‐intensity exercise as compared to a placebo (Bailey et al. 2015).

This study possesses several strengths, including a thorough examination of the literature, extended follow‐up duration compared to prior research, and the assessment of muscle hypertrophy. Nevertheless, it is essential to acknowledge our study's limitations for future research endeavors and the interpretation of our findings. Firstly, our study was open‐label and coincided with the COVID‐19 pandemic, potentially influencing participants' daily routines despite instructions to maintain consistency. Additionally, financial constraints prevented independent verification of active ingredient amounts in supplements provided to athletes. Instead, we relied on a comprehensive literature review to select appropriate doses for desired outcomes. Thirdly, our study did not assess plasma citrulline, arginine, and NO bioavailability, limiting the validation of pharmacokinetic effects. Lastly, our findings pertain specifically to non‐athletic men and may not be extrapolated to the female population.

## 5 Conclusions

In conclusion, watermelon juice supplementation within the initial 24 h post‐exercise may play a physiologically significant role in reducing delayed‐onset muscle soreness, which supports muscle recovery processes. Additionally, regular supplementation over 8 weeks was associated with increased muscle hypertrophy, suggesting that watermelon juice could enhance muscle adaptation to endurance training. These findings highlight watermelon juice as a potential ergogenic aid for individuals in endurance training; however, further studies in diverse populations are warranted to confirm these effects and explore the underlying mechanisms.

## Author Contributions

**Mostafa Norouzzadeh:** conceptualization (lead), investigation (lead), validation (lead), visualization (lead), writing – review and editing (lead). **Behrooz Gholami:** conceptualization (lead), investigation (lead), validation (lead), visualization (lead), writing – review and editing (lead). **Melika Samari:** data curation (equal), writing – original draft (equal). **Hadi Atarod:** methodology (equal), writing – review and editing (equal). **Fatemeh Sadat Hosseini‐Baharanchi:** methodology (equal), writing – review and editing (equal). **Seyedeh Tayebeh Rahideh:** supervision (lead).

## Ethics Statement

The present study followed the guidelines of the Declaration of Helsinki by obtaining written informed consent from participants and received approval from the ethics committee of Iran University of Medical Sciences, Tehran, Iran (Approval number: IR.IUMS.REC.1398.840). Additionally, the trial protocol was registered on the Iranian registry of clinical trials (https://irct.behdasht.gov.ir: IRCT20191105045340N1).

## Consent

The authors have nothing to report.

## Conflicts of Interest

The authors declare no conflicts of interest.

## Supporting information


**Data S1:** fsn371070‐sup‐0001‐DataS1.doc.

## Data Availability

The data sets used and analyzed during the current study are available from the corresponding author on reasonable request.
